# Vaccine Effectiveness Against SARS-CoV-2 Related Hospitalizations in People who had Experienced Homelessness or Incarceration – Findings from the Minnesota EHR Consortium

**DOI:** 10.1007/s10900-023-01308-3

**Published:** 2023-12-08

**Authors:** Malini B. DeSilva, Gregory Knowlton, Nayanjot K. Rai, Peter Bodurtha, Inih Essien, John Riddles, Lemlem Mehari, Miriam Muscoplat, Ruth Lynfield, Elizabeth AK Rowley, Alanna M. Chamberlain, Palak Patel, Alexandria Hughes, Monica Dickerson, Mark G. Thompson, Eric P. Griggs, Mark Tenforde, Tyler NA Winkelman, Gabriela Vazquez Benitez, Paul E. Drawz

**Affiliations:** 1 Health Partners Institute, 8170 33rd Ave South, Mail stop 21112R, Bloomington, MN 55440-1524 USA; 2 Division of Nephrology & Hypertension, University of Minnesota, Minneapolis, MN USA; 3 Health, Homelessness and Criminal Justice Lab, Hennepin Healthcare Research Institute, Minneapolis, MN USA; 4 Westat, Rockville, MD USA; 5 Division of Infectious Disease, Epidemiology, Prevention, and Control, Department of Health, St Paul, Minnesota, MN USA; 6 Minnesota Department of Health, St Paul, MN USA; 7 Mayo Clinic, Rochester, MN USA; 8 Centers for Disease Control and Prevention COVID-19 Response Team, Atlanta, GA USA; 9 General Internal Medicine, Department of Medicine, Hennepin Healthcare, Minneapolis, MN USA

**Keywords:** COVID-19, Homelessness, Minnesota, COVID-19 Vaccines

## Abstract

COVID-19 disproportionately affects people experiencing homelessness or incarceration. While homelessness or incarceration alone may not impact vaccine effectiveness, medical comorbidities along with social conditions associated with homelessness or incarceration may impact estimated vaccine effectiveness. COVID-19 vaccines reduce rates of hospitalization and death; vaccine effectiveness (VE) against severe outcomes in people experiencing homelessness or incarceration is unknown. We conducted a retrospective, observational cohort study evaluating COVID-19 vaccine VE against SARS-CoV-2 related hospitalization (positive SARS-CoV-2 molecular test same week or within 3 weeks prior to hospital admission) among patients who had experienced homelessness or incarceration. We utilized data from 8 health systems in the Minnesota Electronic Health Record Consortium linked to data from Minnesota’s immunization information system, Homeless Management Information System, and Department of Corrections. We included patients 18 years and older with a history of experiencing homelessness or incarceration. VE and 95% Confidence Intervals (CI) against SARS-CoV-2 hospitalization were estimated for primary series and one booster dose from Cox proportional hazard models as 100*(1-Hazard Ratio) during August 26, 2021, through October 8, 2022 adjusting for patient age, sex, comorbid medical conditions, and race/ethnicity. We included 80,051 individuals who had experienced homelessness or incarceration. Adjusted VE was 52% (95% CI, 41–60%) among those 22 weeks or more since their primary series, 66% (95% CI, 53–75%) among those less than 22 weeks since their primary series, and 69% (95% CI: 60–76%) among those with one booster. VE estimates were consistently lower during the Omicron predominance period compared with the combined Omicron and Delta periods. Despite higher exposure risk, COVID-19 vaccines provided good effectiveness against SARS-CoV-2 related hospitalizations in persons who have experienced homelessness or incarceration.

**Supplementary Information:**

The online version contains supplementary material available at 10.1007/s10900-023-01308-3.

## Introduction

Infections with SARS-CoV-2, the virus that causes COVID-19, have disproportionately impacted people experiencing homelessness or incarceration [1–5]. In one study, persons with a recent history of homelessness were > 20 times more likely to be admitted to hospital for COVID-19, > 10 times more likely to require intensive care for COVID-19 and > 5 times more likely to die within 21 days of their first positive COVID-19 test result [6]. The reasons for these disparities in COVID-19 are multifactorial including being housed in congregate settings with the potential for frequent high-risk exposures, potential for limited access to health care services, and the presence of certain underlying medical conditions making them particularly vulnerable to COVID-19 complications [7]. COVID-19 vaccines significantly reduce rates of hospitalization and death for those infected with SARS-CoV-2, however COVID-19 vaccine effectiveness (VE) against severe outcomes in people experiencing homelessness or incarceration has not been evaluated.

During the initial rollout of COVID-19 vaccines, states made individual decisions about prioritization of settings and groups at high-risk for adverse outcomes. One study from the Minnesota Electronic Health Record Consortium (MNEHRC) found that by the end of 2021, completion of COVID-19 primary vaccine series among people experiencing homelessness or incarceration lagged the general population by over 30% [8]. Other studies have also found low rates of COVID-19 vaccination among people experiencing homelessness in several regions in the United States [9]. While homelessness or incarceration alone may not impact vaccine effectiveness, medical comorbidities along with social conditions associated with homelessness or incarceration may impact estimated vaccine effectiveness.

In this evaluation, we aimed to determine COVID-19 vaccine effectiveness against SARS-CoV-2 related hospital admissions in people who had experienced homelessness or incarceration during the combined Delta and Omicron variant predominant periods (August 26, 2021, through October 8, 2022) and Omicron predominant period only (January 1, 2022, through October 8, 2022) and compare these estimates to a demographically and medically comparable population using data from the MNEHRC.

## Methods

### Study Design and Setting

The MNEHRC is a statewide public health–health system collaboration in Minnesota [10, 11]. Eight health systems participated in this MNEHRC cohort study. The MNEHRC data model requires individual level data to be retained in each health system. A distributed code was used to generate each of the steps of the analysis. VE was evaluated in both people who had experienced homelessness or incarceration in the prior year and in a propensity-matched cohort from the general population. This cohort study followed the Strengthening the Reporting of Observational Studies in Epidemiology (STROBE) reporting guideline [12]. Investigators and institutional review boards at each of the participating health systems reviewed and approved the project or deemed it to be public health surveillance [13, 14]. This activity was conducted consistent with applicable federal law and CDC policy (45 C.F.R. part 46.102(l) [2], 21 C.F.R. part 56; 42 U.S.C. Section 241(d); 5 U.S.C. Sect. 552a; 44 U.S.C. Section 3501 et seq.).

### Data Sources

Each MNEHRC health system maintains site-specific common data files. The files consist of weekly records (Sunday through Saturday) that include individuals who experience one or more of the following events in a given week: (1) SARS-CoV-2 or influenza virus test, (2) receipt of a COVID-19 vaccine, or (3) medical encounter (both inpatient and outpatient) with a billing code indicating possible COVID-like-illness. The common data files also include information on whether an individual experienced homelessness or incarceration within the prior year. Each row in the common data file is dedicated to an individual and includes the following data: age, sex, race/ethnicity, SARS-CoV-2 and influenza testing date and result, COVID-19 vaccination date and type, specific comorbid medical conditions, and neighborhood level characteristics (i.e., social vulnerability index and rurality) at the zip code level [15–17]. Because patients may be seen at multiple health systems, each patient is assigned to one health system based on the following: availability of race/ethnicity data, intensity of care in the last 12 and 36 months (defined by number of blood pressures documented in the patient record), and year of last diagnosis in the EHR. Each health system’s site-specific common data files include all patients seen at that system along with indicators for which patients were uniquely assigned to that system.

All sites also create weekly hospitalization files which include all patient admissions. The hospitalization file includes admission and discharge dates, and a link to the site-specific common data file at the patient level. Data on homelessness, incarceration, death, and vaccinations are linked to MNEHRC data using a secure privacy-preserving record linkage (PPRL) process [11, 17].

### Homelessness and Incarceration Data

Data on homelessness came from the Minnesota Homeless Management Information System which captures information regarding use of homeless services [8]. Data on incarceration came from records maintained by the Minnesota Department of Corrections for county jails and state prisons in Minnesota [8]. For this analysis, we used a one year lookback period for both homelessness and incarceration indexed to the most recent receipt of data prior to study period start (i.e., 7/31/2022).

### Death Data

Death data for all-cause mortality came from the Minnesota Department of Health vital statistics reflecting provisional records January 1, 2020 to August 3, 2022.

### COVID-19 Vaccination Data

The Minnesota Immunization Information Connection (MIIC), the state immunization information system, collects information on most vaccines administered in Minnesota. MIIC data provided to the MNEHRC included COVID-19 vaccine week of administration and manufacturer.

### Study Population

Each health system constructed patient cohorts for two time periods: (1) combined Delta and Omicron variant period (August 26, 2021, through October 8, 2022) and (2) Omicron variant period (January 1, 2022, through October 8, 2022). Patients 18 years and older at the start of each variant period were included. Each health system’s cohort included patients uniquely assigned to that health system as well as patients with a hospitalization at that health system during the outcome period. We first identified people who had experienced homelessness or incarceration. After identifying this cohort, we created a 1:3 matched cohort of individuals without a history of homelessness or incarceration but with similar sociodemographics, neighborhood characteristics, and comorbid medical conditions using propensity score matching, as detailed below.

### Outcomes

The primary outcome of interest was a a positive SARS-CoV-2 molecular test the same week as or in the 3 weeks prior to a hospital admission (i.e., “SARS-CoV-2 related hospitalization) [17, 18]. Hospitalizations were evaluated during the combined Delta and Omicron period (August 26, 2021, through October 8, 2022) and during the Omicron only period (January 1, 2022, through October 8, 2022).

### Exposure

At the time of this analysis, CDC recommended that all persons aged ≥ 12 years receive a third dose (booster) of an mRNA vaccine ≥ 5 months after receipt of the second mRNA vaccine dose. Because of this, vaccination status was classified into four categories as follows: (1) completion of primary series only with between 2 and 21 weeks (~ 5 months) since receipt of the last dose, (2) completion of primary series only with at least 22 weeks since receipt of the last dose, (3) completion of primary series and a single booster dose with at least 1 week since receipt of the booster dose, and (4) no prior COVID-19 vaccines. Individuals who had only received one dose of a two dose primary series were excluded consistent with other VE analyses [19].

In the primary analysis, we defined the primary vaccine series as a single adenovirus (AD26.COV.2.S [Janssen/J&J]) vaccine or two of the same mRNA vaccine (BNT162b2 [Pfizer-BioNTech] or mRNA-1273 [Moderna]). A booster dose was defined as an mRNA vaccine following any primary series or an additional adenovirus vaccine following an adenovirus vaccine primary series. Individuals with heterologous vaccine series (e.g., a first dose with mRNA-1273 vaccine followed by a BNT162b2 vaccine) were excluded. In the secondary analysis, we defined the primary series as two of the same mRNA vaccine, and a booster dose as a third dose of the same mRNA primary series.

### Covariates

We included patient age, sex, race/ethnicity, social vulnerability index, and presence of comorbid medical conditions. Patients are assigned a social vulnerability index based on their zip code.

Comorbid medical conditions are defined by the presence of 2 or more ICD-10 codes assigned during medical encounters at any time from January 1, 2016 until a patient experiences an event resulting in inclusion in the site-specific common data files.

### Statistical Analysis

Each health system performed analyses on their own patient cohorts with subsequent pooling of results. Patients alive at the start of the variant period (August 26, 2021, for the Delta and Omicron period, and January 1, 2022, for the Omicron only period) were followed based on their vaccine status until the occurrence of a SARS-CoV-2 related hospitalization, death, end of the study period (October 8, 2022), or a change in their vaccine status. Receipt of a vaccine during the outcome period would result in a change in vaccination status and a new interval would begin 2 weeks after vaccine administration. Patients receiving more than one booster dose were censored at the time of the second booster.

For each time period, a patient cohort with characteristics similar to those of the population that experienced homelessness or incarceration was identified at each health care system through propensity score matching. The propensity score was derived from a logistic regression model that included covariates for age at the start of the variant period, sex, race/ethnicity, substance use disorder (SUD; defined by use of opioids, amphetamine, cocaine, or alcohol), asthma, cancer, COPD, HIV, social vulnerability index (SVI) quartile, and vaccination status at the start of the variant period and homelessness or incarceration as the outcome variable. Exact matching was performed with a 1:3 ratio on 20 strata based on quantiles of the cohort propensity-score distribution [20, 21]. Health system specific data were pulled, and sociodemographic characteristics, comorbidities, and neighborhood characteristics are reported for the population that had experienced homelessness or incarceration and the matched cohort.

Hazard ratios (HR) were estimated at each health care system using a time-dependent Cox proportional hazard model with unvaccinated as the reference group. HRs were estimated as crude associations and after covariate adjustment. Covariates included were age, sex, number of underlying medical conditions, and race/ethnicity. VE and 95% Confidence Intervals (CI) were estimated as 100*(1-HR). Crude and adjusted VE were estimated at each health care system for each cohort, vaccination group, variant period, and for the secondary mRNA restricted analysis. VE estimates across sites were pooled using a random effects meta-analysis model utilizing the generic inverse variance method [22]. All analyses were conducted using R software (4.1.2 version).

## Results

### Study Population

Before matching, there were 4,434,608 eligible persons; including 80,051 identified as having experienced homelessness or incarceration (Table 1). Compared to those who had not experienced homelessness or incarceration, those who had were younger and had a smaller female proportion (28.3% vs. 53.6%). A higher percentage of Minnesotans who had experienced homelessness or incarceration were Black, Hispanic, and American Indian or Alaska Native, and had an address in a zip code designated as a highly vulnerable neighborhood (SVI quartile 1) compared with those who had not experienced homelessness or incarceration. Compared to those who had not experienced homelessness or incarceration, those who had experienced homelessness or incarceration had lower overall vaccination rates (28.6% vs. 61.8%) and among those vaccinated, a higher percentage received a J&J vaccine (28% vs. 8%). After matching, there were fewer differences between the two populations (Table 1).

**Table 1:** Baseline characteristics of the overall population, those with a history of experiencing homelessness or incarceration, those with no history of experiencing homelessness or incarceration, and the matched cohort without history of experiencing homelessness or incarceration for the Delta/Omicron variant period (August 26, 2021, through October 8, 2022) from the Minnesota EHR Consortium

	Overall	History of experiencing homelessness or incarceration	No history of experiencing homelessness or incarceration	Matched Cohort without history of experiencing homelessness or incarceration
**N**	4,434,608	80,051	4,354,557	240,153
**Age Group**				
18–44	1,877,848 (42.3)	56,764 (70.9)	1,821,084 (41.8)	166,948 (69.5)
45–64	1,348,607 (30.4)	20,417 (25.5)	1,328,190 (30.5)	53,482 (22.3)
65–74	648,047 (14.6)	2,439 (3)	645,608 (14.8)	13,168 (5.5)
75–84	369,848 (8.3)	378 (0.5)	369,470 (8.5)	4,781 (2)
85+	190,258 (4.3)	53 (0.1)	190,205 (4.4)	1,774 (0.7)
**Female**	2,353,879 (53.1)	22,670 (28.3)	2,331,209 (53.6)	67,994 (28.3)
**Number of Comorbidities**				
0	2,687,628 (60.6)	53,392 (66.7)	2,634,236 (60.5)	159,905 (66.6)
1	684,645 (15.4)	8,997 (11.2)	675,648 (15.5)	25,535 (10.6)
2+	610,827 (13.8)	5,440 (6.8)	605,387 (13.9)	16,083 (6.7)
Missing	451,508 (10.2)	12,222 (15.3)	439,286 (10.1)	38,630 (16.1)
**Race/Ethnicity**				
American Indian/Alaska Native	47,003 (1.1)	6,132 (7.7)	40,871 (0.9)	11,835 (4.9)
Asian/Pacific Islander	191,949 (4.3)	1,981 (2.5)	189,968 (4.4)	6,109 (2.5)
Black	290,504 (6.6)	17,139 (21.4)	273,365 (6.3)	49,623 (20.7)
Hispanic	189,526 (4.3)	5,290 (6.6)	184,236 (4.2)	17,596 (7.3)
Multiracial	41,359 (0.9)	1,761 (2.2)	39,598 (0.9)	5,099 (2.1)
Other/Unknown/Missing	182,452 (4.1)	3,325 (4.2)	179,127 (4.1)	11,391 (4.7)
White	3,491,815 (78.7)	44,423 (55.5)	3,447,392 (79.2)	138,500 (57.7)
**SVI Quartile**				
1	1,149,701 (25.9)	36,100 (45.1)	1,113,601 (25.6)	105,306 (43.8)
2	874,958 (19.7)	17,762 (22.2)	857,196 (19.7)	54,703 (22.8)
3	857,727 (19.3)	11,641 (14.5)	846,086 (19.4)	36,022 (15)
4	1,021,628 (23)	9,905 (12.4)	1,011,723 (23.2)	30,233 (12.6)
Missing	530,594 (12)	4,643 (5.8)	525,951 (12.1)	13,889 (5.8)
**Rurality**				
Urban	2,716,747 (61.3)	48,419 (60.5)	2,668,328 (61.3)	144,402 (60.1)
Exurban	215,445 (4.9)	3,308 (4.1)	212,137 (4.9)	10,151 (4.2)
Small town	409,313 (9.2)	9,885 (12.3)	399,428 (9.2)	31,099 (12.9)
Rural	559,175 (12.6)	13,639 (17)	545,536 (12.5)	40,174 (16.7)
Missing	530,594 (12)	4,643 (5.8)	525,951 (12.1)	13,889 (5.8)
**Initial Vaccination**				
Janssen/J&J	232,744 (5.2)	6,446 (8.1)	226,298 (5.2)	19,291 (8)
Moderna/Pfizer	2,482,599 (56)	16,436 (20.5)	2,466,163 (56.6)	47,690 (19.9)
Unvaccinated	1,719,265 (38.8)	57,169 (71.4)	1,662,096 (38.2)	173,172 (72.1)
**Substance Use Disorder**	263,598 (5.9)	14,860 (18.6)	248,738 (5.7)	33,389 (13.9)
**Asthma**	227,838 (5.7)	4,123 (6)	223,715 (5.7)	10,074 (5)
**Cancer**	238,487 (5.9)	844 (1.2)	237,643 (6)	4,458 (2.2)
**COPD**	259,633 (6.5)	3,870 (5.6)	255,763 (6.5)	10,204 (5)
**HIV**	6,778 (0.2)	416 (0.6)	6,362 (0.2)	803 (0.4)

Note. N (%); SVI, social vulnerability index; COPD, chronic obstructive pulmonary disease; HIV, human immunodeficiency virus

Among individuals who had experienced homelessness or incarceration, unvaccinated individuals were younger, more likely to be Black or Hispanic Minnesotans, and more likely to live in a rural community compared with unvaccinated individuals who had experienced homelessness or incarceration (Table 2). Individuals who received a booster were older and had more comorbidities compared with those who were unvaccinated or had only received a primary series.

**Table 2:** Patient characteristics by time-varying vaccination category in persons who had experienced homelessness or incarceration for the combined Delta/Omicron variant period (August 26, 2021 - October 8, 2022) from the Minnesota EHR Consortium

	Unvaccinated	Primary Series < 22 weeks	Primary Series ≥ 22 weeks	Booster
**N**	55,896	19,468	22,042	11,872
**Age Group**				
18–44	43,392 (77.6)	12,404 (63.7)	13,143 (59.6)	6,191 (52.1)
45–64	11,474 (20.5)	6,232 (32)	7,478 (33.9)	4,646 (39.1)
65–74	890 (1.6)	745 (3.8)	1,186 (5.4)	874 (7.4)
75–84	124 (0.2)	84 (0.4)	204 (0.9)	140 (1.2)
85+	16 (0)	3 (0)	31 (0.1)	21 (0.2)
**Female**	16,165 (28.9)	5,815 (29.9)	6,525 (29.6)	3,378 (28.5)
**Number of Comorbidities**				
0	38,813 (69.4)	12,029 (61.8)	13,351 (60.6)	6,740 (56.8)
1	5,682 (10.2)	2,703 (13.9)	3,140 (14.2)	1,840 (15.5)
2+	2,911 (5.2)	1,928 (9.9)	2,336 (10.6)	1,528 (12.9)
Missing	8,490 (15.2)	2,808 (14.4)	3,215 (14.6)	1,764 (14.9)
**Race/Ethnicity**				
American Indian/Alaska Native	4,040 (7.2)	1,749 (9)	1,966 (8.9)	1,213 (10.2)
Asian/Pacific Islander	1,186 (2.1)	689 (3.5)	731 (3.3)	367 (3.1)
Black	12,641 (22.6)	3,934 (20.2)	4,168 (18.9)	2,178 (18.3)
Hispanic	3,811 (6.8)	1,237 (6.4)	1,328 (6)	693 (5.8)
Multiracial	1,223 (2.2)	455 (2.3)	513 (2.3)	279 (2.4)
Other/Unknown/Missing	2,574 (4.6)	573 (2.9)	673 (3.1)	340 (2.9)
White	30,421 (54.4)	10,831 (55.6)	12,663 (57.4)	6,802 (57.3)
**SVI Quartile**				
1	25,213 (45.1)	8,860 (45.5)	9,818 (44.5)	5,474 (46.1)
2	12,210 (21.8)	4,518 (23.2)	5,131 (23.3)	2,675 (22.5)
3	8,198 (14.7)	2,804 (14.4)	3,223 (14.6)	1,626 (13.7)
4	6,540 (11.7)	2,564 (13.2)	3,047 (13.8)	1,567 (13.2)
Missing	3,735 (6.7)	722 (3.7)	823 (3.7)	530 (4.5)
**Rurality**				
Urban	32,534 (58.2)	12,978 (66.7)	14,478 (65.7)	7,981 (67.2)
Exurban	2,505 (4.5)	650 (3.3)	743 (3.4)	358 (3)
Small town	6,937 (12.4)	2,245 (11.5)	2,649 (12)	1,342 (11.3)
Rural	10,074 (18)	2,837 (14.6)	3,306 (15)	1,635 (13.8)
Missing	3,735 (6.7)	722 (3.7)	823 (3.7)	530 (4.5)
**Initial Vaccination**				
Janssen/J&J	0 (0)	1,017 (5.2)	2,339 (10.6)	2,742 (23.1)
Moderna/Pfizer	0 (0)	11,908 (61.2)	14,674 (66.6)	7,754 (65.3)
Unvaccinated	55,896 (100)	6,543 (33.6)	5,029 (22.8)	1,376 (11.6)
**Substance Use Disorder**	9,783 (17.5)	4,341 (22.3)	4,668 (21.2)	2,693 (22.7)
**Asthma**	3,102 (6.3)	1,687 (9.5)	1,884 (9.4)	1,111 (10.3)
**Cancer**	459 (0.9)	386 (2.2)	509 (2.5)	358 (3.3)
**COPD**	2,516 (5.1)	1,394 (7.9)	1,664 (8.3)	1,047 (9.7)
**HIV**	236 (0.5)	205 (1.2)	223 (1.1)	178 (1.6)

Note. N (%); SVI, social vulnerability index; COPD, chronic obstructive pulmonary disease; HIV, human immunodeficiency virus

**Fig. 1:**
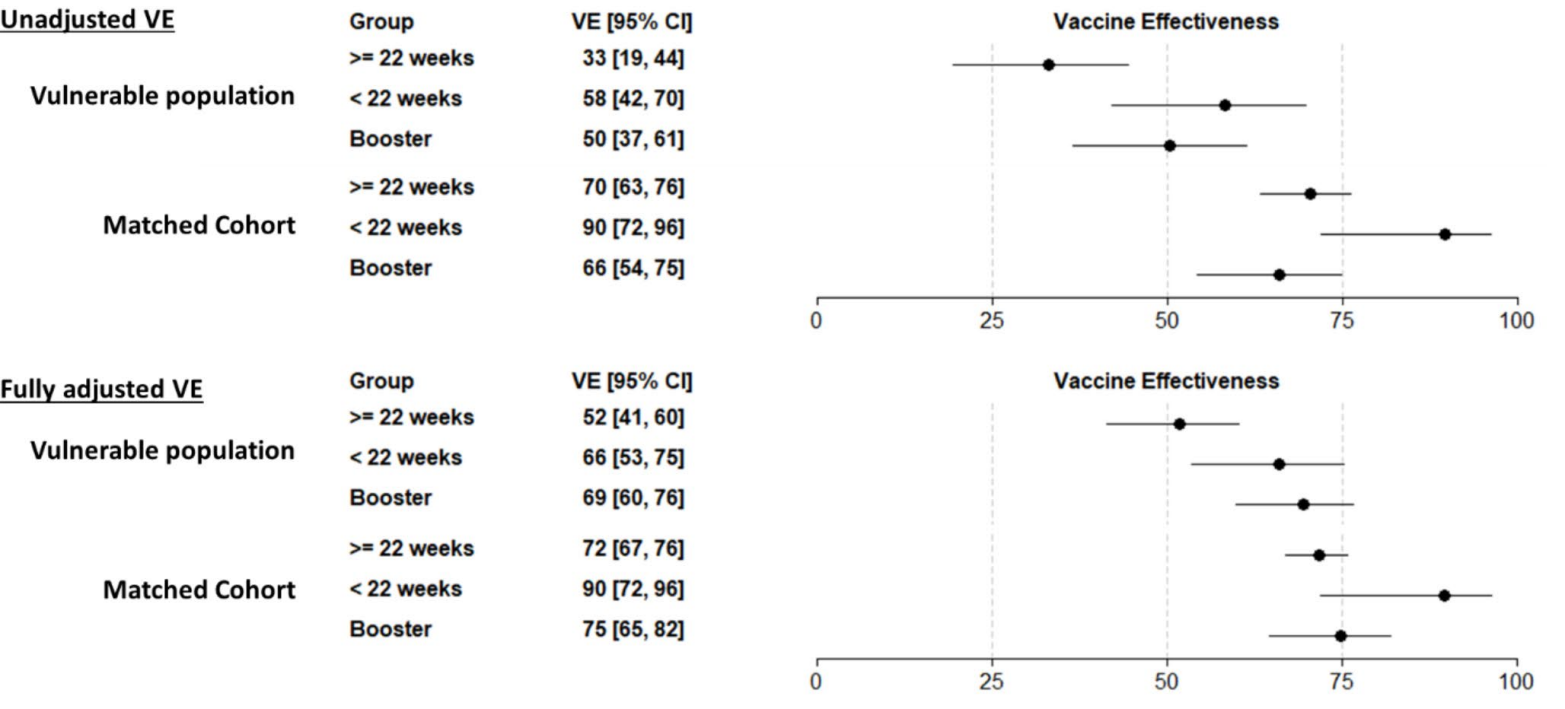
Vaccine effectiveness for COVID-19 hospitalization during the Delta and Omicron periods among populations who had and had not experienced homelessness or incarceration. Note: VE estimates reflect valid combinations of J&J, Pfizer, and Moderna vaccines. In the adjusted models, VE is adjusted by age group, sex, race/ethnicity, and number of underlying medical conditions

Over the combined Delta and Omicron periods, there were 1,095 SARS-CoV-2 related hospitalizations among persons who had experienced homelessness or incarceration and 2,727 among the matched cohort of individuals who had not experienced homelessness or incarceration (Table 3). Adjusted VE in persons who had experienced homelessness or incarceration was 52% (95% confidence interval [CI]:41–60%) among those who were 22 weeks or more past completion of their primary vaccine series, 66% (95% CI: 53–75%) among those who were within 21 weeks of completing their primary vaccine series, and 69% (95% CI: 60–76%) among those who had received a booster (Fig. 1). In persons who had not experienced homelessness or incarceration, point estimates for VE were higher in all vaccine groups compared to persons who had experienced homelessness or incarceration. Results were similar in secondary analyses restricted to those individuals who received mRNA vaccines (Supplemental Fig. 1).

**Table 3:** Number of individuals at risk and number of SARS-CoV-2 related hospitalizations among individuals who had experienced homelessness or incarceration and a matched cohort from the Minnesota EHR Consortium

Population	Vaccination Group	Individuals	SARS-CoV-2 related hospitalizations
**Delta and Omicron Periods**			
Experienced homelessness or incarceration	Unvaccinated	55,896	847 (1.52%)
	Primary ≥ 22 weeks	22,042	133 (0.60%)
	Primary < 22 weeks	19,468	45 (0.23%)
	Boosted	11,872	70 (0.59%)
Matched cohort	Unvaccinated	162,023	2,313 (1.43%)
	Primary ≥ 22 weeks	77,260	181 (0.23%)
	Primary < 22 weeks	62,798	74 (0.12%)
	Boosted	40,690	159 (0.39%)
**Omicron Period**			
Experienced homelessness or incarceration	Unvaccinated	49,309	411 (0.83%)
	Primary ≥ 22 weeks	15,849	86 (0.54%)
	Primary < 22 weeks	9,962	26 (0.26%)
	Boosted	11,288	58 (0.51%)
Matched cohort	Unvaccinated	141,593	1,097 (0.77%)
	Primary ≥ 22 weeks	42,168	109 (0.26%)
	Primary < 22 weeks	19,687	28 (0.14%)
	Boosted	40,708	109 (0.27%)

**Fig. 2:**
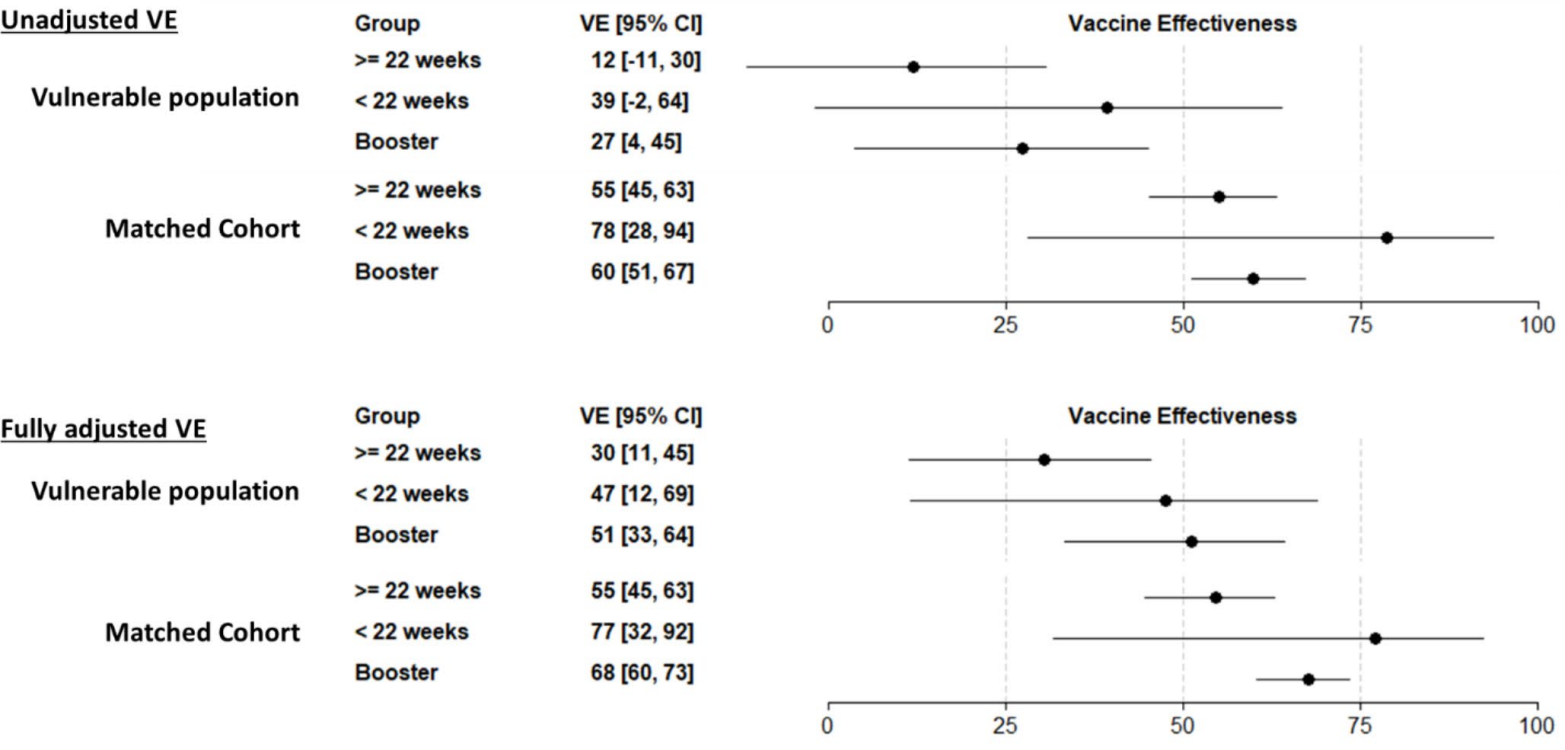
Vaccine effectiveness for COVID-19 hospitalization during the Omicron period among populations who had and had not experienced homelessness or incarceration. Note: VE estimates reflect valid combinations of J&J, Pfizer, and Moderna vaccines. In the adjusted models, VE is adjusted by age group, sex, race/ethnicity, and number of underlying medical conditions

During the Omicron period, adjusted VE in persons who had experienced homelessness or incarceration was 30% (95% CI: 11–45%) in those 22 weeks or more since receiving their primary vaccination, 47% (95% CI: 12–69%) in those less than 22 weeks since receiving their primary vaccination, and 51% (95% CI: 33–64%) in those who had received a booster (Fig. 2). VE point estimates were higher in all groups in the matched cohort of persons who had not experienced homelessness or incarceration.

## Discussion

In this evaluation of over 80,000 persons who had experienced homelessness or incarceration, COVID-19 vaccines provided protection against SARS-CoV-2 related hospitalizations. Point estimates for VE were lower for persons who had experienced homelessness or incarceration compared with a matched cohort who had not experienced homelessness or incarceration. This is noteworthy given the potential for higher exposure in the group who had experienced homelessness or incarceration. Strategies to improve vaccination rates in individuals who have experienced homelessness or incarceration is important because they are less likely to be vaccinated and also at elevated risk for severe COVID-19 due to higher levels of comorbidities and living conditions that make social distancing difficult.

Compared to individuals who had not experienced homelessness or incarceration, individuals who had, were generally younger and more commonly male, Black, Indigenous, living in the most vulnerable SVI quartile, and had a higher percentage of persons with SUD. After propensity score matching to identify a cohort of individuals with similar demographic and medical comorbid conditions who had not experienced homelessness or incarceration, SUD remained slightly higher in the group that had experienced homelessness or incarceration. One report evaluating COVID-19 breakthrough infections in vaccinated persons with SUD compared to persons without SUD found a higher risk of breakthrough infections in those with SUD [23]. Additionally, individuals with SUD who had SARS-CoV-2 breakthrough infections had higher rates of associated hospitalizations and death. Another report found that, among patients with COVID-19, individuals with alcohol use disorder (AUD) were more likely to have a COVID-19 associated hospitalization and higher all-cause mortality than those without AUD [24]. Nevertheless, in our cohort of persons who had experienced homelessness or incarceration, many with a history of SUD, COVID-19 vaccines provided protection against SARS-CoV-2 related hospitalizations.

Given the higher risk for infection in people experiencing homelessness or incarceration, COVID-19 vaccinations are a key infection prevention strategy. Prior to a time of widespread vaccine availability, people experiencing homelessness in Los Angeles County had nearly two times the rates of COVID-19 associated mortality than the general Los Angeles population [25]. Despite this, over 70% of individuals who had experienced homelessness or incarceration in our study were unvaccinated compared to 38% of individuals who had not experienced homelessness or incarceration. This disparity in vaccine coverage highlights the need for tailored strategies to promote COVID-19 vaccination.

Another recent study comparing COVID-19 hospitalizations for people experiencing homelessness or incarceration to the general U.S. population found higher frequencies of hospitalization, readmission, and longer lengths of stay in people experiencing homelessness or incarceration [2]. These results highlight the importance of efforts to reduce risk for COVID-19 related outcomes in these populations. This study relied on ICD-10 and admission codes to identify people experiencing homelessness or incarceration [2]. The MNEHRC’s ability to integrate health system data with state databases is a strength of our study and expands our ability to identify persons who fall into vulnerable categories. It allows for statewide population-based estimates of disease and provides health systems and public health organizations in Minnesota the ability to perform surveillance in specific risk groups with the goal of offering targeted risk mitigation strategies. It is reassuring that our study demonstrated good COVID-19 VE in those who had experienced homelessness or incarceration, especially after a booster dose.

Our study is subject to the following limitations. First, our definitions for people who have experienced homelessness or incarceration may overestimate the number of individuals currently in those populations, as we use a one year look back period for inclusion. Further, for people experiencing homelessness, we are only focused on those who have had encounters with housing services in the past year and therefore lack information about unsheltered people experiencing homelessness which may result in these individuals being classified as being part of the general population. Second, we do not account for the impact of natural infection or outpatient antiviral treatment on VE in our analysis. Third, we do not evaluate the relative VE of various vaccination strategies, rather we compare all vaccination statuses to unvaccinated. Fourth, events were defined as a hospitalization within 3 weeks before or 1 week after the date of admission and do not include any diagnostic codes assigned during the hospitalization. This may have resulted in capture of admissions where COVID-19 was identified incidentally and not related to the reason for admission. Given that SARS-CoV-2 infection is more common among persons experiencing homelessness or incarceration than the general population, this may have biased VE downward in this group [26]. Fifth, due to limited precision, VE was combined across variant periods and vaccine product types in the primary analysis and vaccination exposure groups were limited (e.g., they did not account for time since vaccination among those who received a vaccine booster dose). Sixth, our study population was limited to people with medical encounters at a participating health system or who received a COVID-19 vaccine. While this covers the majority of people in the State of Minnesota, there may be populations that are underrepresented in our data, including those whose seek care at federally qualified health centers, due to low frequency of contact with participating health systems or who did not receive a COVID-19 vaccine.

In our study, despite lower vaccination rates and potential for higher COVID-19 exposures in people experiencing homelessness or incarceration, COVID-19 vaccines reduced risk for SARS-CoV-2 related hospitalizations. Booster vaccination is important for prevention against severe COVID-19 disease in all populations including those who have experienced homelessness or incarceration. Initiatives to increase vaccination rates may reduce severe outcomes from COVID-19 in those who have experienced homelessness or incarceration.

### Electronic Supplementary Material

Below is the link to the electronic supplementary material.

Supplementary Material 1

## References

[1] Meehan AA, Thomas I, Horter L (2022). Incidence of COVID-19 among persons experiencing homelessness in the US from January 2020 to November 2021. JAMA Netw Open.

[2] Montgomery MP, Hong K, Clarke KEN (2022). Hospitalizations for COVID-19 among US people experiencing incarceration or homelessness. JAMA Netw Open.

[3] Imbert E, Kinley PM, Scarborough A (2021). Coronavirus Disease 2019 outbreak in a San Francisco homeless shelter. Clinical Infectious Diseases.

[4] Reinhart E, Chen DL (2020). Incarceration and its disseminations: COVID-19 pandemic lessons from Chicago’s Cook County Jail. Health Aff (Millwood).

[5] Saloner B, Parish K, Ward JA, DiLaura G, Dolovich S (2020). COVID-19 cases and deaths in federal and state prisons. Journal of the American Medical Association.

[6] Richard L, Booth R, Rayner J, Clemens KK, Forchuk C, Shariff SZ (2021). Testing, Infection and complication rates of COVID-19 among people with a recent history of homelessness in Ontario, Canada: A retrospective cohort study. CMAJ Open.

[7] Winkelman TNA, Phelps MS, Mitchell KL, Jennings L, Shlafer RJ (2020). Physical health and disability among U.S. adults recently on community supervision. Journal of Correctional Health Care: The Official Journal of the National Commission on Correctional Health Care.

[8] Shearer RD, Vickery KD, Bodurtha P (2022). COVID-19 vaccination of people experiencing homelessness and incarceration in Minnesota. Health Aff (Millwood).

[9] Montgomery MP, Meehan AA, Cooper A (2021). Notes from the field: COVID-19 vaccination coverage among persons experiencing homelessness - six U.S. jurisdictions, December 2020-August 2021. Mmwr. Morbidity and Mortality Weekly Report.

[10] Drawz PE, DeSilva M, Bodurtha P (2022). Effectiveness of BNT162b2 and mRNA-1273 second doses and boosters for severe Acute Respiratory Syndrome Coronavirus 2 (SARS-CoV-2) Infection and SARS-CoV-2-related hospitalizations: A statewide report from the Minnesota Electronic Health Record Consortium. Clinical Infectious Diseases.

[11] Winkelman TNA, Margolis KL, Waring S (2022). Minnesota Electronic Health Record Consortium COVID-19 Project: Informing pandemic response through statewide collaboration using observational data. Public Health Reports.

[12] von Elm E, Altman DG, Egger M (2008). The strengthening the reporting of Observational studies in Epidemiology (STROBE) statement: Guidelines for reporting observational studies. Journal of Clinical Epidemiology.

[13] Office of Human Research Protections. Activities Deemed Not to Be Research: Public Health Surveillance 2018 Requirements. US Department of Health and Human Services, Washington, DC (Nov 2018). https://www.hhs.gov/ohrp/regulations-and-policy/requests-for-comments/draft-guidance-activities-deemed-not-be-research-public-health-surveillance/index.html. Accessed Dec 21 2021.

[14] National Archives and Records Administration (Accessed Dec 21 2021). Code of Federal Regulations, Title 45. College Park, MD. https://www.ecfr.gov/current/title-45/subtitle-A/subchapter-A/part-46/subpart-A/section-46.102#p-46.102(l).

[15] Centers for Disease Control and Prevention (CDC), Agency for Toxic Substances and Disease Registry (ATSDR). Social Vulnerability Index (Oct 2022). https://www.atsdr.cdc.gov/placeandhealth/svi/index.html. Accessed Oct 13 2021.

[16] US Department of Agriculture, Economic Research Service (2021). Rural-urban Commuting Area codes. Washington, DC. https://www.ers.usda.gov/data-products/rural-urban-commuting-area-codes.aspx. *Accessed)ct* 13.

[17] Winkelman TNA, Rai NK, Bodurtha PJ (2022). Trends in COVID-19 vaccine administration and effectiveness through October 2021. JAMA Netw Open.

[18] Tartof SY, Slezak JM, Fischer H (2021). Effectiveness of mRNA BNT162b2 COVID-19 vaccine up to 6 months in a large integrated health system in the USA: A retrospective cohort study. Lancet.

[19] Tenforde MW, Weber ZA, Natarajan K (2022). Early estimates of Bivalent mRNA vaccine effectiveness in preventing COVID-19-Associated Emergency Department or Urgent Care encounters and hospitalizations among immunocompetent adults - VISION Network, Nine States, September-November 2022. Mmwr. Morbidity and Mortality Weekly Report.

[20] Desai RJ, Rothman KJ, Bateman BT, Hernandez-Diaz S, Huybrechts KF (2017). A propensity-score-based fine stratification approach for confounding adjustment when exposure is infrequent. Epidemiology (Cambridge, Mass.).

[21] Ho D, Imai K, King G, Stuart EA (2011). MatchIt: Nonparametric preprocessing for parametric causal inference. Journal of Statistical Software.

[22] Borenstein M, Hedges LV, Higgins JP, Rothstein HR (2010). A basic introduction to fixed-effect and random-effects models for meta-analysis. Res Synth Methods.

[23] Wang L, Wang Q, Davis PB, Volkow ND, Xu R (2022). Increased risk for COVID-19 breakthrough Infection in fully vaccinated patients with substance use disorders in the United States between December 2020 and August 2021. World Psychiatry.

[24] Bailey KL, Sayles H, Campbell J (2022). COVID-19 patients with documented Alcohol Use Disorder or alcohol-related Complications are more likely to be hospitalized and have higher all-cause mortality. Alcoholism, Clinical and Experimental Research.

[25] Chang AH, Kwon JJ, Shover CL (2022). COVID-19 mortality rates in Los Angeles County among people experiencing homelessness, March 2020-February 2021. Public Health Reports.

[26] Bowen M, Marwick S, Marshall T (2019). Multimorbidity and emergency department visits by a homeless population: A database study in specialist general practice. British Journal of General Practice.

