# Supplementary material for: Vaccine Effectiveness Against SARS-CoV-2 Related Hospitalizations in People who had Experienced Homelessness or Incarceration – Findings from the Minnesota EHR Consortium
Source: J Community Health. 2023 Dec 8;49(3):448–57. doi: 10.1007/s10900-023-01308-3 (PMC10981627; doi:10.1007/s10900-023-01308-3)

Supplemental Figure 1. Vaccine effectiveness during Delta and Omicron periods among individuals who had experienced homelessness or incarceration in the primary analyses (J&J, Pfizer, and Moderna vaccines) (top) and secondary analyses (only Pfizer and Moderna vaccines) (bottom). VE is adjusted by age group, sex, race/ethnicity, and number of underlying medical conditions.


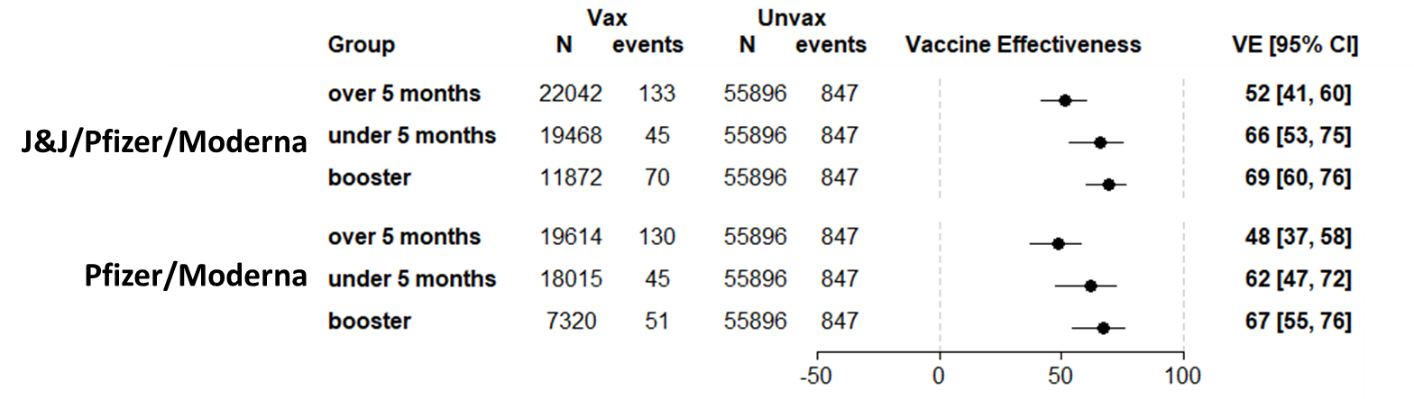

Supplement: Supplementary file 1 — Supplementary Material 1 [file 10900_2023_1308_MOESM1_ESM.docx]
